# Infant nutrition and risk of celiac disease

**DOI:** 10.1186/1824-7288-40-S1-A42

**Published:** 2014-08-11

**Authors:** Carlo Catassi

**Affiliations:** 1 Department of Pediatrics, Università Politecnica delle Marche, Ancona, Italy

## 

Celiac disease (CD) is an immune-mediated enteropathy triggered by the ingestion of gluten in genetically susceptible individuals. Gluten is the major protein component of wheat, rye and barley. The major predisposing genes are the HLA-DQ2 and DQ8 genotypes found in at least 95 % of patients. CD is one of the most common lifelong disorders on a worldwide basis affecting 0.5-1 % of the general population in Western countries. CD is a multifactorial disorder that depends on both genetic and environmental factors for expression.

The analysis of factors associated with an epidemic of early-onset CD in Sweden during the 1980-90s indicated a role for infant nutrition, the disease risk being substantially lower in infants introducing small amount of gluten when still breast fed . The protective role of breast feeding has been supported by other case-control studies. As far as weaning, an increased risk of CD has been reported in infants introducing gluten containing food before the age of 4 months. Most of these data investigated only clinically detected cases of CD, leaving unanswered the question whether factors related to infant protect or simply delay the onset of disease. Ongoing European, prospective studies on at-risk infants followed on by serological screening are shedding light on the role of infant nutrition in the development of CD. The Italian Baby Study recently completed a 10-years period of follow-up. The results of this study suggest that genetically predisposed children tend to develop CD autoimmunity early in life, generally before the age of 5 years. Early dietary factors, particularly age at gluten introduction, seem to play a minor role in the development of CD, at least in infants at family risk, questioning the validity of the “window of exposure” hypothesis (lower risk in infants introducing the new antigen between 4 and 6 months). The possible preventive effect of delayed gluten introduction in infants with high HLA risk (double copy of DQB1*02) is a puzzling finding deserving further investigation. In this study, breast-feeding in children at-risk of CD did not seem to exert any clear-cut effect on the disease risk. Another European, prospective and randomized study ( PreventCD) will soon clarify whether small amount of gluten given during breast feeding do indeed protect from CD development.

